# Experimental Study on a Ceramic Membrane Condenser with Air Medium for Water and Waste Heat Recovery from Flue Gas

**DOI:** 10.3390/membranes11090701

**Published:** 2021-09-13

**Authors:** Da Teng, Liansuo An, Guoqing Shen, Shiping Zhang, Heng Zhang

**Affiliations:** School of Energy, Power and Mechanical Engineering, North China Electric Power University, Beijing 102206, China; zsnjer@163.com (D.T.); liansuoan@ncepu.edu.cn (L.A.); zhangshiping@ncepu.edu.cn (S.Z.); zhangchongheng@ncepu.edu.cn (H.Z.)

**Keywords:** ceramic membrane condenser, water and waste heat recovery, flue gas, native pressure air, sensitivity analysis, moisture content

## Abstract

Ceramic membrane condensers that are used for water and waste heat recovery from flue gas have the dual effects of saving water resources and improving energy efficiency. However, most ceramic membrane condensers use water as the cooling medium, which can obtain a higher water recovery flux, but the waste heat temperature is lower, which is difficult to use. This paper proposes to use the secondary boiler air as the cooling medium, build a ceramic membrane condenser with negative pressure air to recover water and waste heat from the flue gas, and analyze the transfer characteristics of flue gas water and waste heat in the membrane condenser. Based on the experimental results, it is technically feasible for the ceramic membrane condenser to use negative pressure air as the cooling medium. The flue gas temperature has the most obvious influence on the water and heat transfer characteristics. The waste heat recovery is dominated by latent heat of water vapor, accounting for 80% or above. The negative pressure air outlet temperature of the ceramic membrane condenser can reach 50.5 °C, and it is in a supersaturated state. The research content of this article provides a new idea for the water and waste heat recovery from flue gas.

## 1. Introduction

As of 2018, s third of the world’s electricity is supplied by coal, especially in China, where coal-fired power generation accounts for up to 69%. In the electric power market, the coal-fired power plant occupies a crucial position. In addition, the rapid development of steel, cement and other industries also consumes about 80% of the world’s coal resources—the proportion of China’s is about 78% [1]. Coal is an important energy source; thus, it is essential to alleviate a resource shortage by improving the utilization efficiency of coal.

At present, the boiler tail flue gas of a coal-fired power plant is characterized by high moisture content and low temperature [2]. The flue gas was purified by a wet desulfurization tower to remove SO_2_ and reach a wet saturation state. The flue gas temperature is reduced to 55~60 °C, so direct discharge of flue gas will cause a large amount of water and waste heat loss [3]. For instance, in the traditional 330 MW coal-fired power plant, the flue gas temperature at the outlet of the wet desulfurization tower is 55 °C, so the low-temperature waste heat loss caused by the discharge of wet saturated flue gas is 484 GJ/h, which is equivalent to wasting 16 t/h standard coal according to the calorific value. In the meantime, the flue gas contains 120 t/h of water directly discharged into the atmosphere with the flue gas. If the water recovery efficiency from the flue gas is 40–60%, and the condensate water is used as make-up water for the desulfurization tower, then the wet desulfurization system can achieve zero water consumption. By recovering the sensible heat of flue gas and the latent heat of water vapor, the coal-fired power plant thermal efficiency can increase more than 5% [4,5]. In addition, the lower moisture content in the flue gas will help promote the dissipation of pollutants in the flue gas and reduce “gypsum rain” around coal-fired power plants [6]. In summary, water and waste heat recovery play an important role in enhancing energy efficiency, reducing water consumption, and improving the environment of coal-fired power plants.

The water and waste heat loss caused by low-temperature flue gas emission has attracted the attention of a large number of researchers, and a variety of technical solutions [7] have also been proposed for the water and waste heat recovery from flue gas. Common technical methods are condensation [8,9,10], absorption [11,12,13] or membrane separation [14,15]. Condensation method using a recuperative heat exchanger or a spray tower [16,17], the flue gas temperature is reduced to a temperature below the dew point of water vapor. The water vapor in the flue gas is condensed and precipitated to achieve the water and waste heat recovery from the flue gas. The absorption method includes liquid absorption or solid absorption, and the liquid absorption method is more researched. The absorber adopts the spray tower structure to ensure full contact between the low temperature, high concentration dehumidification solution, and the wet saturated flue gas. This solution has the characteristics of low saturated water vapor pressure to absorb moisture and heat in the flue gas, while using high-temperature flue gas to realize the regeneration of low concentration dehumidification solution. Compared with the condensation method, the liquid absorption method has stronger water and waste heat recovery capabilities. This method will face problems, such as contamination of the dehumidifying solution, higher operating costs. The membrane separation method uses a new type of heat exchanger material, which uses different transmission rates of different gases in porous or dense membrane materials to achieve the recovery of clean water from flue gas.

**Figure 1:**
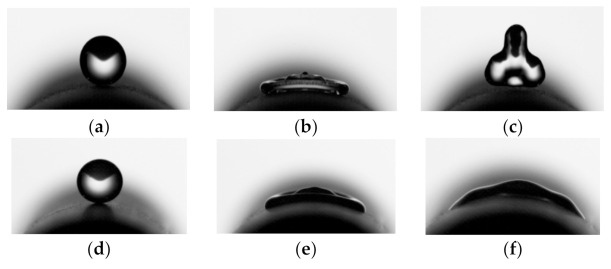
The contact process of droplets on the membrane surface. (**a**) Hydrophobic membrane time: 0 ms contact; (**b**) hydrophobic membrane time: 8 ms spread; (**c**) hydrophobic membrane time: 10.8 ms rebound; (**d**) hydrophilic membrane time: 0 ms contact; (**e**) hydrophilic membrane time: 13.05 ms spread; (**f**) hydrophilic membrane time: 16.85 ms adsorb.

As the core of the membrane separation method for water and waste heat recovery from flue gas, membrane materials can be divided into hydrophobic membranes or hydrophilic membranes [18,19,20]. As shown in Figure 1, as captured with a high-speed camera, droplets falling on the surface of the hydrophobic ceramic membrane will rebound and will eventually bounce off the surface of the ceramic membrane. However, the droplets falling on the surface of the hydrophilic ceramic membrane will cause adsorption, and will quickly penetrate into the ceramic membrane material. As far as the hydrophobic membrane is concerned, the moisture on the flue gas side forms a droplet condensation on the surface of the hydrophobic membrane, which prevents moisture in the flue gas from passing through the membrane material, but allows other gases in the flue gas to pass through the membrane material. Brunetti et al. [14] have used hollow fiber membrane (PVDF) to make a hydrophobic membrane condenser and applied it in the wet saturated flue gas dehydration process. Through the interception effect of the hydrophobic hollow fiber membrane, a water recovery efficiency of 20–25% can be obtained. Compared with hydrophobic membranes, hydrophilic membranes have been studied and applied more. The moisture on the flue gas side forms film condensation on the surface of the hydrophilic membrane. Liquid water on the membrane surface or membrane pores can prevent other gases from entering the flue gas. Only water in the flue gas should be permitted to pass through the membrane material. 

As early as 2000, the Natural Gas Technology Institute (GTI) of the United States successfully developed TMC technology [21] based on hydrophilic nanoporous ceramic membranes under the auspices of the United States Department of Energy (DOE). At the same time, the hydrophilic ceramic membrane that uses circulating water as the cooling medium has a high water and heat recovery capacity. Since then, according to the application performance of the hydrophilic porous ceramic membrane, many scholars have carried out a series of research and discussion on key influencing factors such as flue gas flow, flue gas temperature, cooling water flow, cooling water temperature and membrane pore size [4,5,22]. In addition, Hu Haowei et al. [23] compared the performance of hydrophilic and hydrophobic nanoporous ceramic composite membranes in water and waste heat recovery in the mixed gas of nitrogen and water vapor, and found that the water and heat recovery of hydrophilic membranes is better than that of hydrophobic membranes.

**Figure 2:**
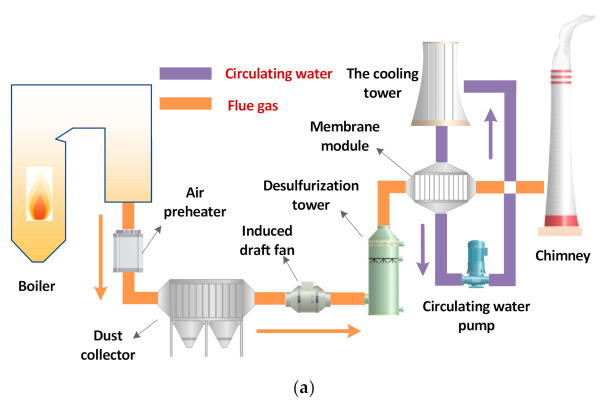

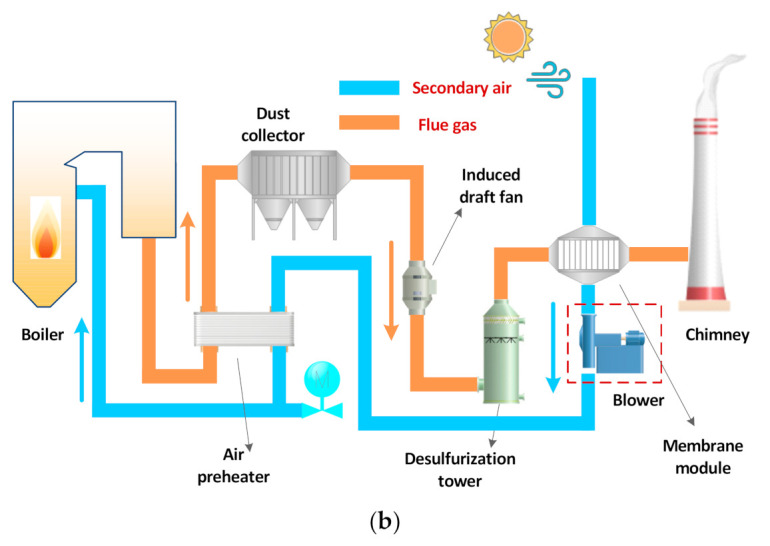
Application of ceramic membrane condenser in coal-fired power plant. (**a**) Circulation water cooling medium. (**b**) Secondary air cooling medium.

In previous studies [24,25,26,27,28], this research group also used circulating water as the cooling medium for water and waste heat recovery by ceramic membrane condenser. Figure 2a shows the water and waste heat recovery application system under the full flue gas volume of a coal-fired power plant. The tail flue gas of the coal-fired power plant boiler is discharged from the air preheater, the dust collector removes the solid dust particles in the flue gas, the wet desulfurization tower absorbs the acid gas in the flue gas, and finally, the flue gas is directly emission after recovering part of the water and waste heat through the ceramic membrane condenser. After the circulating water in the ceramic membrane condenser absorbs the water and waste heat from flue gas, the temperature continues to rise. In addition, the water temperature needs to be kept stable through the cold water tower, and then the ceramic membrane condenser continues to circulate. However, the low temperature of circulating water makes its heat unable to be effectively used.

Based on previous research, this paper proposes another idea, which is to use negative pressure air as the cooling medium. As shown in Figure 2b, the gas pipeline connects the inlet of the secondary fan from the ceramic membrane condenser. The negative pressure air absorbs the water and waste heat from flue gas and is introduced as the secondary air of the boiler. Compared with circulating water as the cooling medium, negative pressure air has a lower ability to recover the water and waste heat from flue gas, but can realize the effective utilization of low-temperature waste heat of the flue gas, and can also inhibit the formation of high-temperature nitrogen oxides in the furnace.

## 2. Method and Experiments System

### 2.1. Membrane Materials

**Figure 3:**
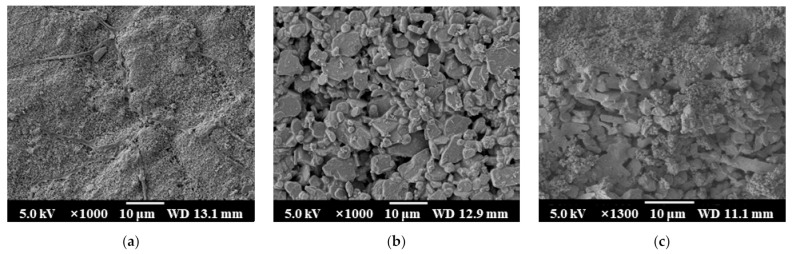

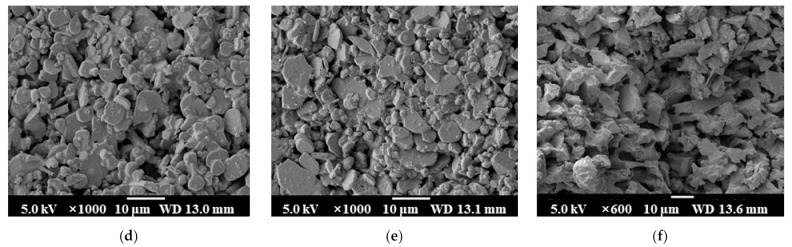
SEM image of ceramic membrane sample. (**a**) 10 nm membrane outside surface; (**b**) 10 nm membrane inside surface; (**c**) 10 nm membrane truncation surface; (**d**) 1 µm membrane outside surface; (**e**) 1 µm membrane inside surface; (**f**) 1 µm membrane truncation surface.

**Figure 4:**
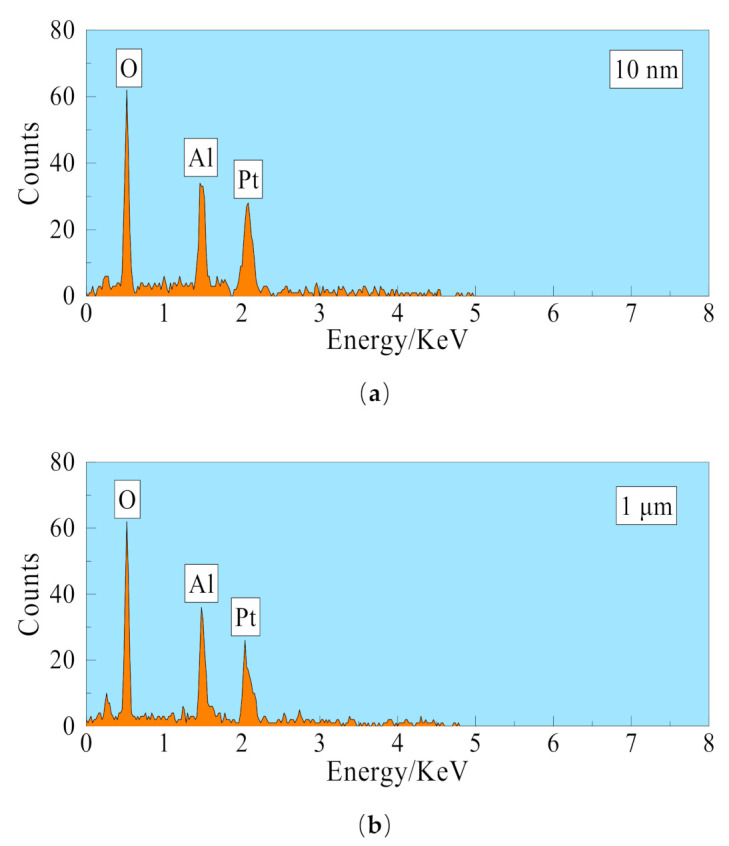
Energy-dispersive X-ray spectroscopy spectra of ceramic membrane sample. (**a**) 10 nm ceramic membrane. (**b**) 1 μm ceramic membrane.

As shown in Figure 3a–c, the outer surface pore size of the 10 nm ceramic membrane is significantly smaller than the inner surface pore size of the 10 nm ceramic membrane, and the cross-section has an obvious layered structure. The 10 nm ceramic membrane is an asymmetric structure [27]. As shown in Figure 3d–f, the inner and outer surface pore size of the 1 μm ceramic membrane are basically the same in this experiment, and there is no obvious layered structure in the cross-section. The 1-μm ceramic membrane is a symmetrical structure membrane [27]. The symmetrical membrane production process is relatively simple, and the production cost is relatively low. As shown in Figure 4a,b, 10 nm ceramic membrane and 1 μm ceramic membrane after qualitative analysis by EDS (Energy Dispersive Spectroscopy), the main elements in the membrane material are O element and Al element. The Pt element in the picture is introduced by spraying gold before the test. It is confirmed that the membrane material is inorganic alumina. The ceramic membranes used in this experiment are all tubular membranes. The specific structural parameters are shown in Table 1. They are divided into four different pore sizes of 0.4 nm, 10 nm, 100 nm, and 1 μm. Except for the 1-μm ceramic membrane, which is a symmetrical membrane; the rest are asymmetric membranes. The porosity of the four types of ceramic membranes is basically the same. Moreover, the other structural parameters of the ceramic membrane are the same, with the length of 790 mm, inner diameter of 8 mm, and wall thickness of 2 mm. The ceramic membrane module shell is made of stainless steel (AISI 316L) and its length is 800 mm, inner diameter 20 mm, and wall thickness is 1 mm. The ceramic membrane module is insulated with insulation materials.

### 2.2. System Overview

**Figure 5:**
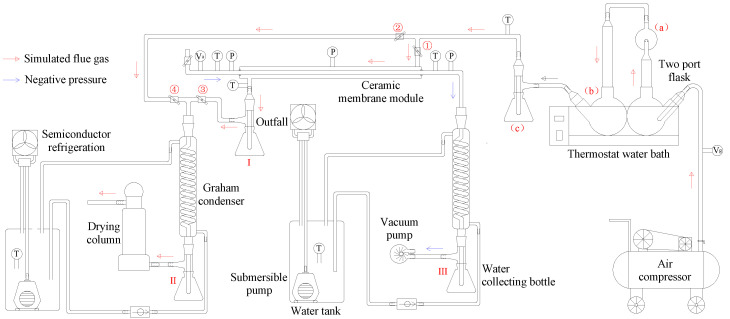
Ceramic membranes water and waste heat recovery from simulate flue gas experiment platform.

The inorganic ceramic membrane condenser using negative pressure air as a cooling medium experimental system is shown in Figure 5. The experimental system is mainly composed of a flue gas generation subsystem, a ceramic membrane module subsystem, a flue gas drying subsystem, a semiconductor refrigeration subsystem, and a negative pressure air condensing subsystem. The simulated dry flue gas in the flue gas generation subsystem is continuously and stably supplied by an air compressor. The dry flue gas is measured by a metal tube float flowmeter and then enters a two-necked flask in a constant temperature water bath. The flask contains quantitative desulfurization wastewater after pretreatment, which is used to achieve heating and humidification of dry flue gas to wet saturation. The membrane module subsystem has a sleeve structure, and the wet saturated flue gas enters the shell side of the membrane module, and the negative pressure air enters the tube side of the membrane module. The flue gas and airflow in the opposite direction for heat and mass transfer in the membrane module. The flue gas at the outlet of the ceramic membrane module is condensed by the serpentine condenser and is dried by the drying tower, finally is discharged directly to the atmosphere. At the same time, the flue gas drying subsystem can also be used to verify the supersaturation coefficient of the simulated flue gas. The semiconductor refrigeration subsystem is made by semiconductor modules, and is used to make low-temperature circulating water which is used for flue gas or negative pressure air condensation. The air condensing subsystem uses air as a cooling medium to absorb water and waste heat from flue gas. The inlet of the vacuum pump is connected with the outlet of the serpentine condenser to provide a certain negative pressure environment. The water collection device is used to collect negative pressure air condensate, so as to effectively measure the amount of recovered water.

By controlling the opening and closing state of the valve in the experimental system, the experimental process is mainly divided into two stages:

(1) When valves 2, 4 are opened, and 1, 3 are closed, the wet flue gas at the outlet of the flue gas generation subsystem directly enters the flue gas drying subsystem. The flue gas drying subsystem measures the volume of condensed water in the water collection bottle II and the weight gain of the drying tower under different flue gas flow, and then determines the supersaturation coefficient of the wet flue gas in the experimental system.
(2) When valves 1, 3 are opened and 2, 4 are closed, the wet flue gas at the outlet of the flue gas generation subsystem enters the shell side of the ceramic membrane module, and a series of flue gas water and waste heat recovery experiments are carried out.

Table 2 shows the range of operating parameters selected during the experiment. The negative pressure air temperature is relatively stable, while the flue gas temperature, flue gas flow, and airflow have a wide range.

As shown in Table 3, multiple types of data acquisition instruments are arranged in the experimental platform. These instruments are used to measure pressure, temperature and other parameters in real-time. The inlet and outlet of the membrane module tube side are respectively arranged with temperature and pressure monitoring points. The middle position of the membrane module shell side is arranged with pressure measuring points. The inlet and outlet of the membrane module shell side are respectively arranged with temperature measuring points. A metal rotor flowmeter is arranged at the outlet of the air compressor. A glass rotameter is arranged at the negative pressure air inlet of the membrane module tube side. And two water tanks are arranged with temperature measuring points.

### 2.3. Heat and Mass Transport Model

**Figure 6:**
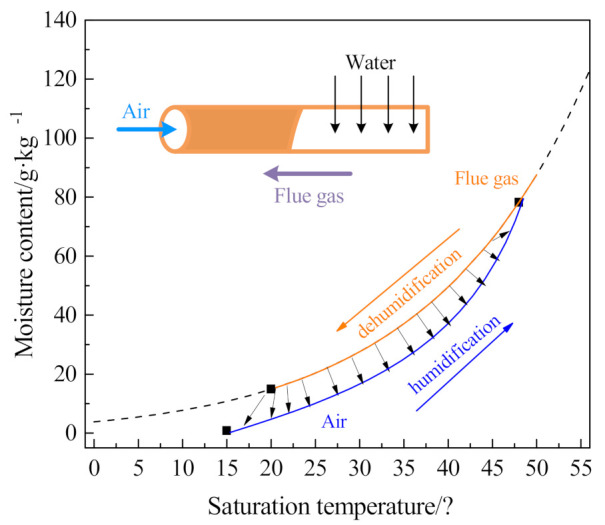
Mass and heat transfer process in the ceramic membrane.

As shown in Figure 6, the simulated flue gas outside the ceramic membrane is wet and saturated; however, the negative pressure air inside the ceramic membrane has a relatively low relative humidity. The two flow in the ceramic membrane module in the reverse direction, and simultaneously carry out the transfer of water and waste heat [29]. The moisture content of the flue gas in the ceramic membrane module shell side gradually decreases along the moisture saturation line, which is a cooling and dehumidification process. While the temperature and moisture content of negative pressure air in the ceramic membrane module tube side continue to rise, which is a heating and humidification process, and it may eventually reach a moisture saturation state [20].

**Figure 7:**
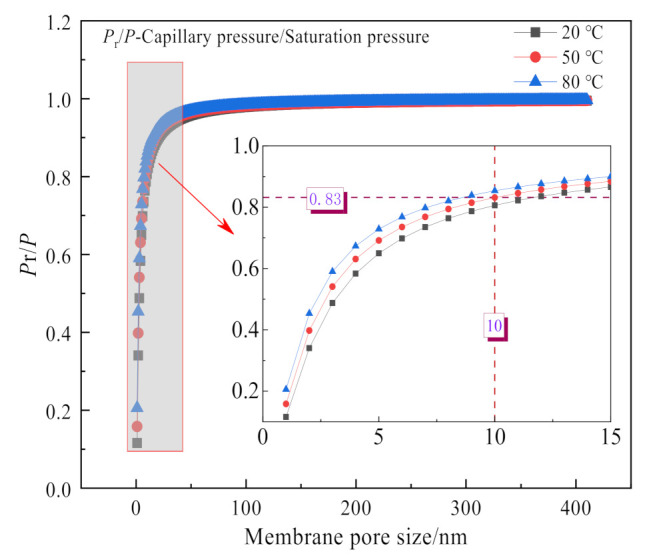
Capillary condensation pressure in the pore size.

**Figure 8:**
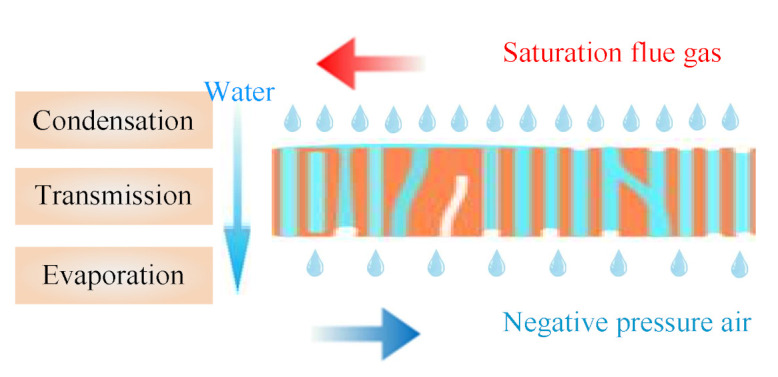
The water in flue gas transport process.

The inorganic ceramic membrane is a porous material. According to the Kelvin equation, the water vapor in the flue gas will undergo capillary condensation in the porous material below the saturated vapor pressure [27,30,31]. As shown in Figure 7, the smaller the membrane pore size, the more obvious the capillary phenomenon. At the same time, combined with the membrane pore size selected, it can be considered that capillary condensation will occur within the pore size of 0.4 nm and 10 nm ceramic membranes, while capillary phenomenon will not occur in 100 nm and 1 μm pore size ceramic membranes [2,32]. Considering that the tube size and shell size of the ceramic membrane module are gas-phase fluids, Figure 8 shows that the mass transfer process of flue gas moisture across the membrane can be roughly divided into three steps [33]: the condensation process on the outer surface of the membrane, the transfer process within the membrane pores, and the evaporation process on the inner surface of the membrane. Because the outer surface of the membrane is coated with a hydrophilic selective separation layer, the condensation process is mainly cooling condensation and film condensation [18]. When the membrane pore size is smaller, capillary condensation will occur due to capillary forces, which will help the outer surface of the ceramic membrane to condense [6]. The liquid water in the membrane pores is transported from the outer surface of the ceramic membrane to the inner surface of the ceramic membrane under the pressure difference. The tube size of the ceramic membrane module is negative pressure air. Under the conditions of relative humidity and negative pressure, liquid water on the inert surface of the ceramic membrane continuously evaporates into the negative pressure air. At this time, when the membrane pore size is smaller, the capillary force will hinder the evaporation of water.

### 2.4. Methods

#### 2.4.1. Supersaturation Coefficient

In order to accurately determine the recovery characteristics of the water and waste heat from flue gas by the ceramic membrane, it is necessary to clearly simulate flue gas inlet parameters. Therefore, it is necessary to accurately determine the moisture content of the flue gas at the outlet of the flue gas generation subsystem. The flue gas supersaturation coefficient can be shown as the following formula:

(1) $$\alpha =\frac{1000V^{*}\rho_{w}+V_{g}\Delta t\rho_{g}d_{T_{o}^{g}}}{V_{g}\Delta t\rho_{g}d_{T_{i}^{g}}}$$

where α is the supersaturation coefficient of wet flue gas, $V^{*}$ is the condensate volume of bottle II, mL, $\rho_{w}$ is the water density, 1 g/mL, $V_{g}$ is the dry flue gas flow (151 kPa, 20 °C), L/min, Δt is run time, min, $\rho_{g}$ is the dry flue gas density (151 kPa, 20 °C), 1.80 kg/m^3^, $d_{T_{o}^{g}}$ is the saturation moisture content of flue gas outlet, g/kg, $d_{T_{i}^{g}}$ is the saturation moisture content of flue gas inlet, g/kg.

#### 2.4.2. Water Recovery Characteristics

The serpentine condenser uses low-temperature cold water to cool the negative pressure air at the outlet of the ceramic membrane module. And the collection bottle III measures the volume of condensed water. Therefore, the water recovery rate and water recovery efficiency from flue gas are as follows [34,35]:

(2) $$\upsilon =\frac{3V}{50S\Delta t}$$

(3) $$\beta =\frac{1000\rho_{w}V}{\Delta tV_{g}\rho_{g}\alpha d_{T_{i}^{g}}}\times 100$$

where υ is the water recovery rate, L/(h·m^2^), V is the condensate volume of bottle III, mL, S is the outside surface area of the ceramic membrane, 297.67 × 10^−4^ m^2^, β is the water recovery efficiency, %.

#### 2.4.3. Heat Recovery Characteristics

Ceramic membrane module and corresponding pipelines are insulated, so the heat dissipation loss is negligible. The flue gas at the inlet of the ceramic membrane module is wet saturation. The waste heat of flue gas mainly includes two parts: sensible heat of flue gas and latent heat of water vapor [5]. The waste heat recovery power and waste heat recovery efficiency from flue gas are shown in the following formula [27,36]:

(4) $$Q_{S}=\frac{V_{g}\rho_{g}\left[\left({\alpha d}_{T_{i}^{g}}-d_{T_{o}^{g}}\right)\left(T_{i}^{g}-T_{o}^{a}\right)C_{p}^{w}+1000\left(T_{i}^{g}-T_{o}^{g}\right)C_{p}^{g}+\left(T_{i}^{g}-T_{o}^{g}\right)d_{T_{o}^{g}}C_{p}^{\mathrm{gw}}\right]}{{6\times 10}^{7}}$$

(5) $$Q_{L}=\frac{V_{g}\rho_{g}\left(d_{T_{i}^{g}}-d_{T_{o}^{g}}\right)r}{{6\times 10}^{4}}$$

(6) $${Q=Q}_{L}+Q_{S}$$

(7) ηS=QSQ; ηL=QLQ

(8) $$\eta =\frac{6\times 10^{9}Q}{V_{g}\rho_{g}\left[1000rd_{T_{i}^{g}}+\alpha d_{T_{i}^{g}}\left(T_{i}^{g}-T_{e}\right)C_{p}^{w}+1000\left(T_{i}^{g}-T_{e}\right)C_{p}^{g}\right]}$$

where $Q_{S}$ is the sensible heat recovery power, W, $Q_{L}$ is the latent heat recovery power, W, Q is heat recovery power, W, $T_{o}^{a}$ is the negative pressure air outlet temperature, °C, $C_{p}^{w}$ is the specific heat capacity of water, 4200J/(kg·K), $C_{p}^{g}$ is the specific heat capacity of dry flue gas, 1007J/(kg·K), $C_{p}^{\mathrm{gw}}$ is the specific heat capacity of water vapor, 1871 J/(kg·K), r is the latent heat of water, 2257 kJ/kg, $\eta_{S}$ is the sensible heat recovery efficiency, %, $\eta_{L}$ is the latent heat recovery efficiency, %, η is the heat recovery efficiency, %, $T_{e}$ is ambient temperature, °C.

#### 2.4.4. Moisture Characteristics of Negative Pressure Air

Using negative pressure air as the cooling medium, it is defined that moisture content and relative humidity of negative pressure air at the outlet of the ceramic membrane module. As shown in the following formula:

(9) $$d_{a}=\frac{1000\rho_{w}V}{\Delta tV_{a}\rho_{a}}$$

(10) $$\varphi =\frac{d_{a}}{d_{T_{o}^{a}}}$$

where $d_{T_{o}^{a}}$ is the saturation moisture content of negative pressure air outlet, g/kg, $V_{a}$ is negative pressure airflow, L/min, $\rho_{a}$ is negative pressure air density, 1.2 kg/m^3^, $d_{a}$ is moisture content of negative pressure air, g/kg, φ is relative humidity of negative pressure air, %.

#### 2.4.5. Sensitivity Analysis

Ceramic membrane condenser uses negative pressure air to achieve water and waste heat recovery from flue gas. There are many factors that affect water and waste heat recovery characteristics, such as flue gas temperature, flue gas flow, negative pressure airflow, and ceramic membrane pore size. Taking *R* = 10 nm, $V_{g}$ = 10.33 L/min, $T_{i}^{g}$ = 56.31 °C, $V_{a}$ = 15 L/min as reference conditions, as shown in the following formula, the various factors and results are dimensionless, and the sensitivity of each factor is analyzed.

(11) $$\Gamma_{n}=\frac{{Ν}_{n}}{{Ν}_{n}^{0}}∼\Pi_{n}^{1}=\frac{v_{n}^{}}{v_{n}^{0}};\Pi_{n}^{2}=\frac{Q_{n}^{}}{Q_{n}^{0}}$$

where Γ is dimensionless coefficient of the independent variable, Ν is independent variable values, $\Pi_{n}^{1}$ is dimensionless coefficient of water recovery, $\Pi_{n}^{2}$ is dimensionless coefficient of heat recovery, n is value 1, 2, 3, 4, respectively represent R, $V_{g}$, $T_{i}^{g}$, $V_{a}$, 0 is the comparative experiment condition (membrane pore size 10 nm, flue gas flow 10.33 L/min, flue gas inlet temperature 56.31 °C, negative pressure airflow 15 L/min).

## 3. Results and Discuss

**Figure 9:**
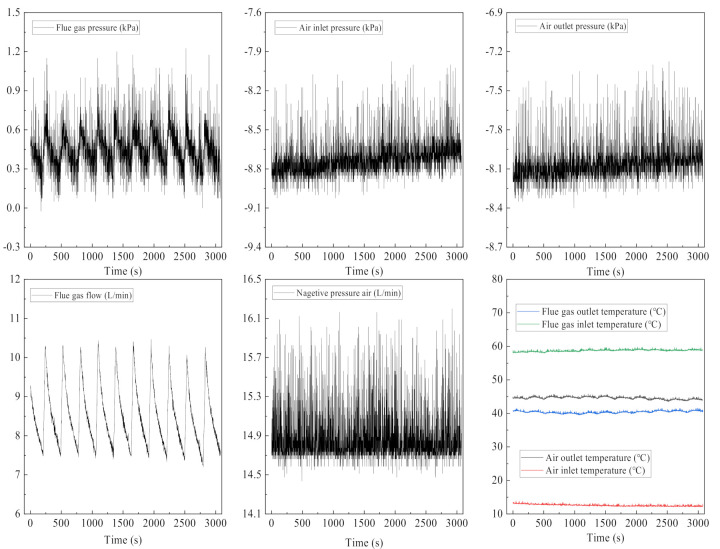
Running state parameters. Experiment conditions: flue gas flow 8.5 L/min, flue gas inlet temperature 58.6 °C, negative pressure airflow 15 L/min, negative pressure air inlet temperature 12.8 °C, membrane pore size 10 nm.

The experimental system runs continuously for 30 min, and the state parameters are shown in Figure 9. The flue gas flow and flue gas pressure show regular fluctuations, which are determined by the working principle of the air compressor. When the air compressor tank pressure is within a certain range, the air compressor is in a stopped state. When the pressure is lower than the set value, the air compressor starts to start. The inlet and outlet pressure of the ceramic membrane module tube side is relatively stable, and the outlet pressure is greater than the inlet pressure. The negative pressure air absorbs water and waste heat from flue gas, leading to an increase in the outlet temperature and contains a lot of water vapor. The negative pressure airflow in the ceramic membrane is relatively stable. The inlet and outlet temperature of the flue gas and negative pressure air in the ceramic membrane module are relatively stable, and the order is $T_{i}^{g}$ > $T_{o}^{a}$ > $T_{o}^{g}$ > $T_{i}^{a}$.

### 3.1. Supersaturation Coefficient of Flue Gas

**Figure 10:**
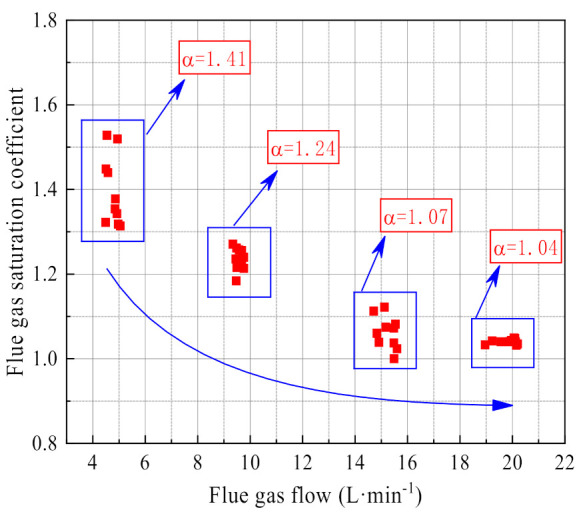
Supersaturation coefficient of flue gas.

As shown in Figure 10, the flue gas supersaturation coefficient changes greatly with the flue gas flow. When the flue gas flow is low, the supersaturation coefficient is higher and fluctuates greatly. When the flue gas flow increases, the flue gas supersaturation coefficient gradually stabilizes and tends to 1. When the flue gas flow is low, the gas-liquid separation device uses the inertia of the airflow to separate the droplets poorly, resulting in the flue gas supersaturation coefficient being too high. When the flue gas flow is 4.75 L/min, 9.47 L/min, 15.2 L/min, and 19.83 L/min, the average flue gas supersaturation coefficients are, respectively, 1.41, 1.24, 1.07, 1.04.

### 3.2. Water and Heat Recovery Characteristics

#### 3.2.1. Effect of Flue Gas Flow

**Figure 11:**
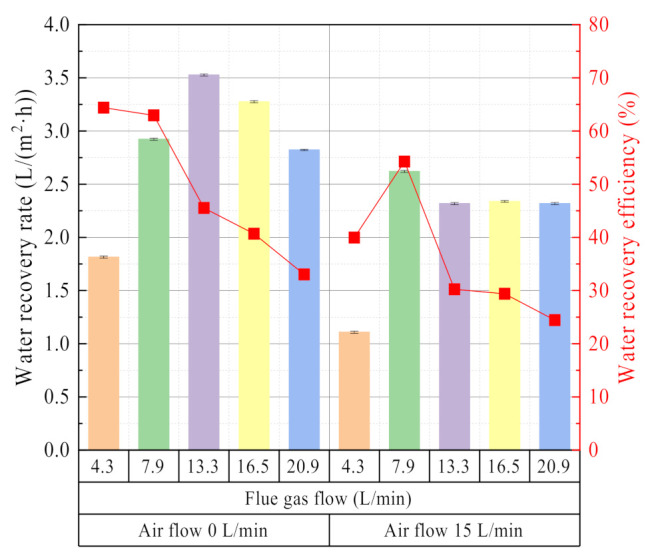
Water recovery rate/efficiency vs. Flue gas flow. Experiment conditions: flue gas inlet temperature 56.36 °C, negative pressure airflow 15 L/min, negative pressure air inlet temperature 14.08 °C, membrane pore size 10 nm.

As shown in Figure 11, the influence of flue gas flow on the characteristics of water and waste heat recovery is divided into two types. One is that the inlet valve of the ceramic membrane module tube side is closed, the glass rotor flowmeter shows zero. The water recovery rate varies with flue gas flow first increases and then decreases, and the water recovery efficiency shows a gradual decline. The outside surface of the ceramic membrane is mainly capillary condensation, and the inner surface of the ceramic membrane is mainly vacuum evaporation. The other is that the inlet valve of the membrane module tube side is fully open, the glass rotameter is 15 L/min. The water recovery rate first increases and then decreases gradually, while the water recovery efficiency first increases and then decreases. At this time, the outer surface of the ceramic membrane is mainly cooled and condensed, and the inner surface of the ceramic membrane is mainly purged and evaporated.

Compared with negative pressure air or circulating water as the cooling medium, there are some differences in water recovery characteristics with the flue gas flow [18,24,27]. With the increase of flue gas flow, the average water vapor pressure increases in the membrane module shell side, and the non-condensable gas boundary layer decreases in the membrane module shell side. Thus, both of the above points can help to improve the water recovery rate from flue gas. However, continuing to increase the flue gas flow correspond to a shorter residence time for water vapor in the membrane module. At the same time, the cooling capacity of the negative pressure air is low, resulting in the water vapor in the flue gas being discharged from the membrane module before it can be recovered. Therefore, the water recovery rate decreases.

**Figure 12:**
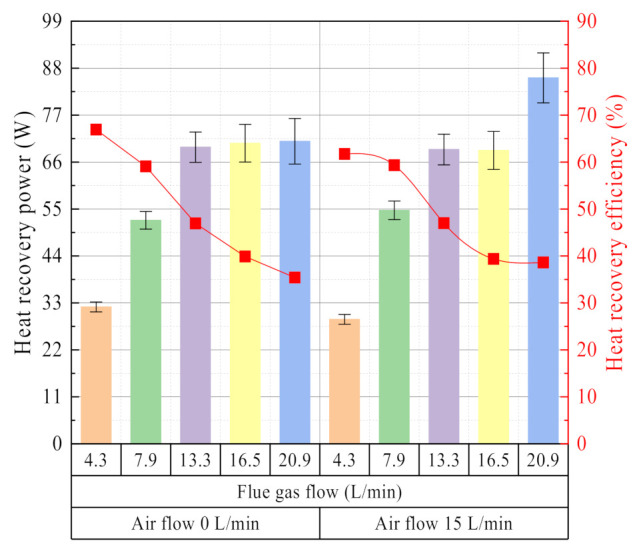
Heat recovery power/efficiency vs. flue gas flow. Experiment conditions: flue gas inlet temperature 56.36 °C, negative pressure airflow 15 L/min, negative pressure air inlet temperature 14.08 °C, membrane pore size 10 nm.

In the above two cases, the effect of flue gas flow on heat recovery characteristics is relatively consistent. As shown in Figure 12, when the flue gas flow increases, the heat recovery power increases, but the heat recovery efficiency decreases. These experimental results are consistent with the literature [27,30,34]. As the flue gas flow increases, it helps to maintain a higher heat exchange temperature difference and reduce the thickness of the non-condensable gas boundary layer in the membrane shell side. Therefore, the heat recovery power rises with the increase of the flue gas flow. However, more heat is discharged out of the ceramic membrane module along with the flue gas before it can be recovered, resulting in a decrease in heat recovery efficiency.

#### 3.2.2. Effect of Flue Gas Temperature

**Figure 13:**
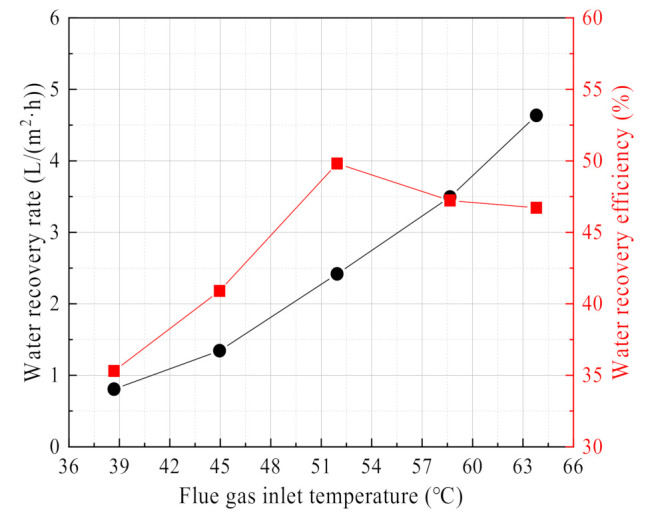
Water recovery rate/efficiency vs. Flue gas temperature. Experiment conditions: flue gas flow 9.85 L/min, negative pressure airflow 15 L/min, negative pressure air inlet temperature 12.95 °C, membrane pore size 10 nm.

Figure 13 shows the change curve of water recovery rate and efficiency with flue gas temperature. The water recovery rate shows a continuously increasing trend with the increase of flue gas temperature, while the water recovery efficiency first increases and then decreases with the increase of flue gas temperature. Compared with the literature [18,33,34,37], the experimental results in this paper show the same regular variation for the water recovery rate, but there are some differences for water recovery efficiency. In the literature [34], the water recovery efficiency increases with the increase of flue gas temperature. In the literature [33], the water recovery efficiency decrease with the increase of flue gas temperature. In the literature [33,34], there are obvious differences in the arrangement and area of the ceramic membrane tubes, leading to different experimental results. Compared with the literature [33,34], the cooling medium is negative pressure air in this paper. When the flue gas temperature increases, the moisture content and water vapor partial pressure increase exponentially in the wet saturated flue gas [18,26,38]. The condensation process on the membrane module shell side, the transport process across the membrane and the evaporation process on the membrane module tube side are all enhanced; thus, the water recovery rate increase. However, the water recovery efficiency is affected by water recovery rate and moisture content of flue gas. When the flue gas temperature is low, increasing the flue gas temperature, the moisture content of flue gas increases slowly, resulting in a growth multiple of the water recovery rate higher than the growth multiple of the moisture content of the flue gas. When the flue gas temperature is high, increasing the flue gas temperature, the moisture content of flue gas increases rapidly. The growth multiple of water recovery rate is lower than the growth multiple of moisture content of flue gas. Therefore, the water recovery efficiency first increases to the maximum value and then decreases.

**Figure 14:**
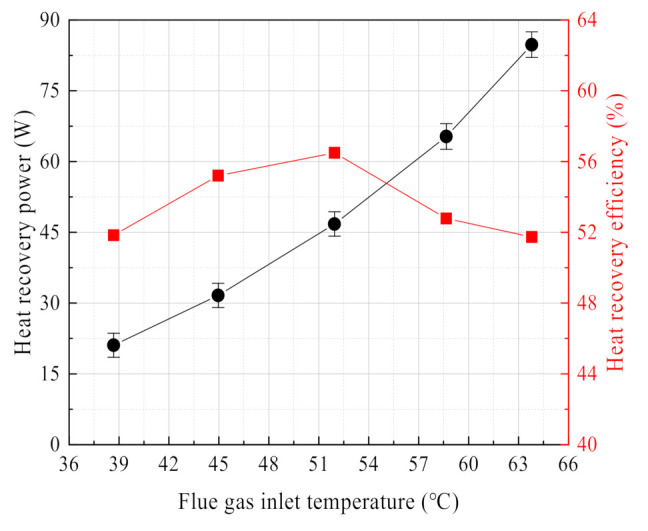
Heat recovery power/efficiency vs. flue gas temperature. Experiment conditions: flue gas flow 9.85 L/min, negative pressure airflow 15 L/min, negative pressure air inlet temperature 12.95 °C, membrane pore size 10 nm.

The flue gas heat recovery power and efficiency show the same change curve as the water recovery characteristics. As shown in Figure 14, as the flue gas temperature increases, the heat recovery power continues to increase. While the heat recovery efficiency first increases and then decreases. Regarding the effect of flue gas temperature on the heat recovery power, the experimental results in this paper are consistent with the literature [18,27,37]. However, regarding the effect of flue gas temperature on the heat recovery efficiency, the experimental results in this paper are somewhat different with the literature [27] because the increase of flue gas temperature helps to increase the heat exchange temperature difference of the membrane module. When the flue gas temperature is higher, the moisture content also increases, and the phase change heat is stronger. However, negative pressure air has a lower cooling capacity compared to the circulating water. After the heat recovery efficiency increases to the maximum, the flue gas temperature continues to increase, which is limited by the remaining fixed experimental parameters, inevitably leading to the heat recovery efficiency to decline.

#### 3.2.3. Effect of Negative Pressure Airflow

**Figure 15:**
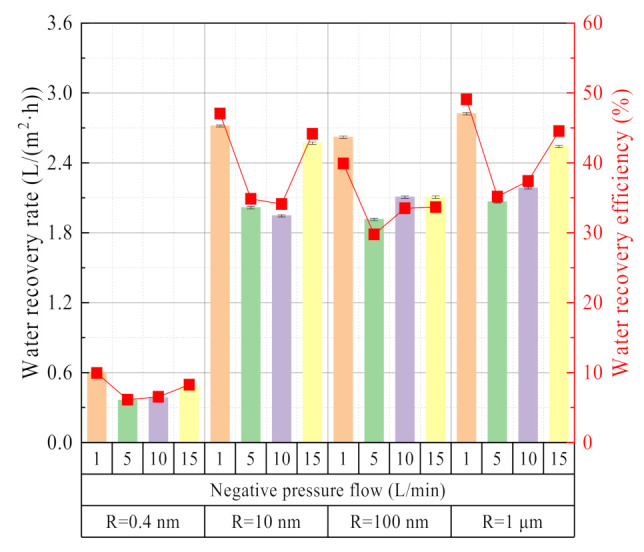
Water recovery rate/efficiency vs. Negative pressure airflow. Experiment conditions: flue gas flow 10.58 L/min, flue gas inlet temperature 56.31 °C, negative pressure air inlet temperature 12.68 °C.

The negative pressure airflow mainly affects the water and waste heat recovery characteristics from two angles: condensation on the outside surface of the ceramic membrane and evaporation on the inside surface of the ceramic membrane. It can be seen from Figure 15 that under any same membrane pore size, as the negative pressure airflow increases, the water recovery rate and efficiency show a trend of first decreasing and then increasing. Basically, when the negative pressure airflow is 5 L/min, the water recovery rate and efficiency are the lowest value. When the negative pressure airflow is 1 L/min, the vacuum degree in the ceramic membrane is higher, which helps the evaporation of liquid water on the inside surface of the membrane. When the negative pressure airflow is 15 L/min, the negative pressure in the ceramic membrane is small. However, the high negative pressure airflow has a strong cooling capacity, which helps the condensation process on the outside surface of the ceramic membrane to occur. When the negative pressure airflow is 5 L/min, it weakens the process of evaporation on the inside surface of the ceramic membrane and condensation on the outside surface of the ceramic membrane and results in the lowest water recovery rate and efficiency. The water recovery rate and efficiency in this paper are much higher than the study results in the literature [6]. The membrane condenser used in this paper is a porous ceramic membrane, while the membrane condenser used in the literature [6] is a dense hollow fiber membrane.

**Figure 16:**
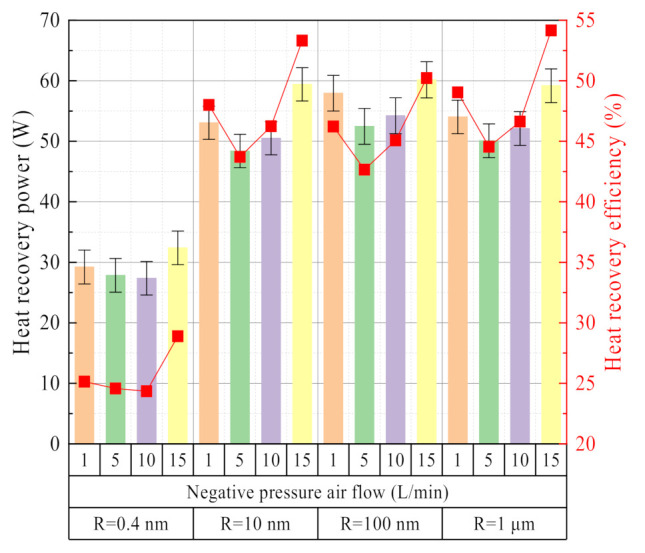
Heat recovery power/efficiency vs. negative pressure airflow. Experiment conditions: flue gas flow 10.58 L/min, flue gas inlet temperature 56.31 °C, negative pressure air inlet temperature 12.68 °C.

The influence of the negative pressure airflow on the heat recovery characteristics is basically the same as the water recovery characteristics. As shown in Figure 16, both of which show a change characteristic of first decreasing and then increasing with the increase of the negative pressure airflow. When the vacuum degree is larger, the heat absorption of liquid water evaporation is dominant by high vacuum. When the negative pressure airflow is large, the heat absorption of liquid water is dominated by relative humidity. At this time, the heat recovery power and efficiency reach the maximum. The inorganic ceramic membrane has a strong coupling and synchronization effect on the water and waste heat recovery characteristics from the flue gas.

#### 3.2.4. Effect of Membrane Pore Size

**Figure 17:**
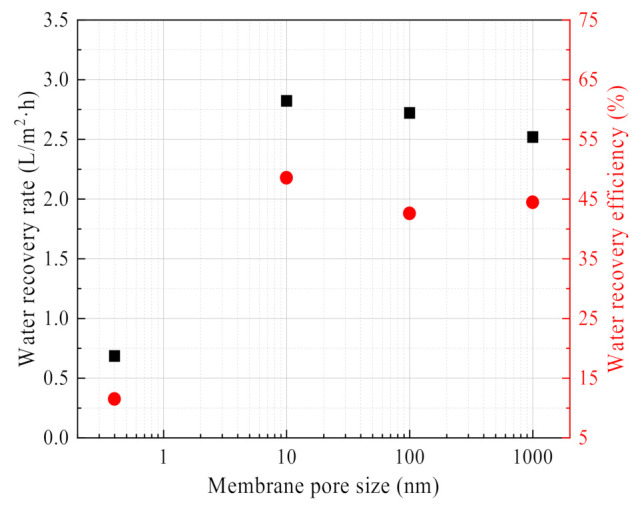
Water recovery rate/efficiency vs. Membrane pore size. Experiment conditions: flue gas inlet temperature 56.27 °C, flue gas flow 10.58 L/min, negative pressure airflow 0 L/min.

From Figure 17, it can be seen that the water recovery rate and efficiency of 0.4 nm ceramic membranes are much lower than that of 10 nm, 100 nm and 1 μm ceramic membranes. The membrane pore size is too small to transport liquid water in the membrane pores. At the same time, 10 nm ceramic membrane water recovery performance is slightly higher than that of the 100 nm, 1 μm ceramic membrane, because capillary condensation occurs on the outside surface of the 10 nm ceramic membrane, which helps to improve the water recovery characteristics from the flue gas. The same finding was also confirmed in the literature [2].

**Figure 18:**
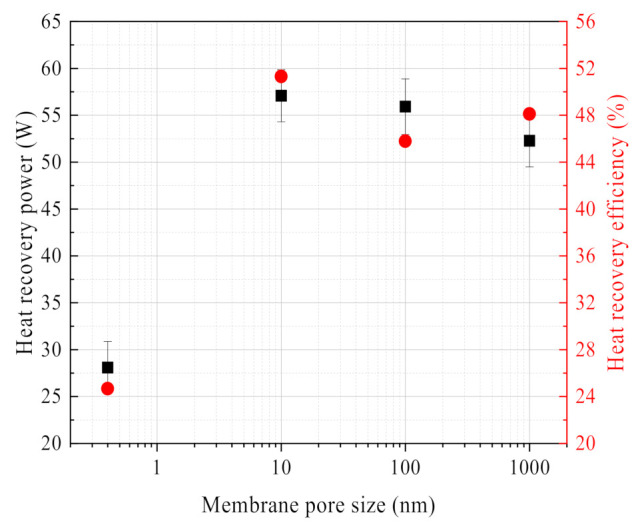
Heat recovery power/efficiency vs. membrane pore size. Experiment conditions: flue gas inlet temperature 56.27 °C, flue gas flow 10.58 L/min, negative pressure airflow 0 L/min.

Comparing the influence of ceramic membrane pore size on the water recovery characteristics, Figure 18 shows that the influence of membrane pore size on waste heat recovery characteristics has a similar rule. The 10 nm ceramic membrane has the highest heat recovery power and efficiency, while the 0.4 nm ceramic membrane has the lowest heat recovery power and efficiency.

#### 3.2.5. The Driving Force for Water and Waste Heat Recovery

**Figure 19:**
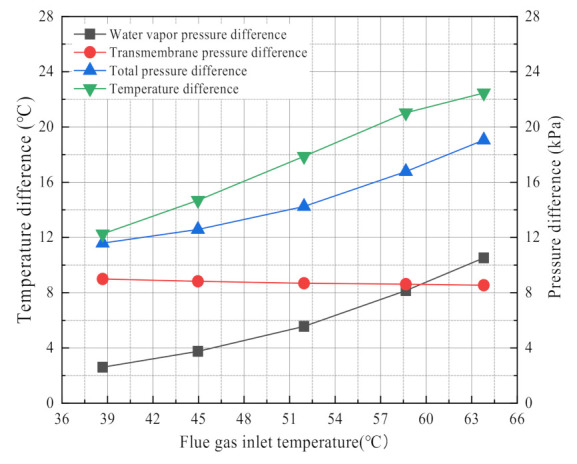
The driving force for moisture and waste heat recovery. Experiment conditions: flue gas flow 9.85 L/min, negative pressure airflow 15 L/min, negative pressure air inlet temperature 12.95 °C, membrane pore size 10 nm.

Many studies have shown that, the major driving force for the waste heat recovery from flue gas is the logarithmic mean temperature difference by membrane condensers [5,30,33,36]. However, the major driving force for the water recovery from flue gas is less analyzed. Many scholars have focused on the water transport processes in membrane materials, such as capillary condensation [2,6,27,39]. In this paper, it is discussed that the driving force of heat and mass transfer in the membrane condenser by using the flue gas temperature as the critical variable. As shown in Figure 19, the water vapor partial pressure difference in the membrane module increases rapidly with the increase of flue gas temperature, while transmembrane pressure difference basically does not change with the flue gas temperature. The water recovery rate also increases with the increase of flue gas temperature. Thus, it can be seen that the water vapor partial pressure difference is the key factor to affect the flue gas moisture recovery rate, while it has little correlation with the transmembrane pressure difference. Likewise, logarithmic mean temperature difference increases with the increase of flue gas temperature, and shows essentially the same trend as the heat recovery power. Therefore, the logarithmic mean temperature difference in the membrane condenser is a key factor for waste heat recovery.

### 3.3. Sensitivity Analysis

**Figure 20:**
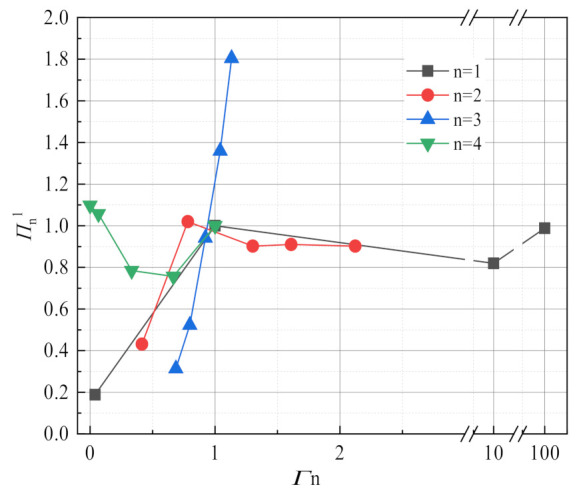
Sensitivity analysis of flue gas water recovery.

**Figure 21:**
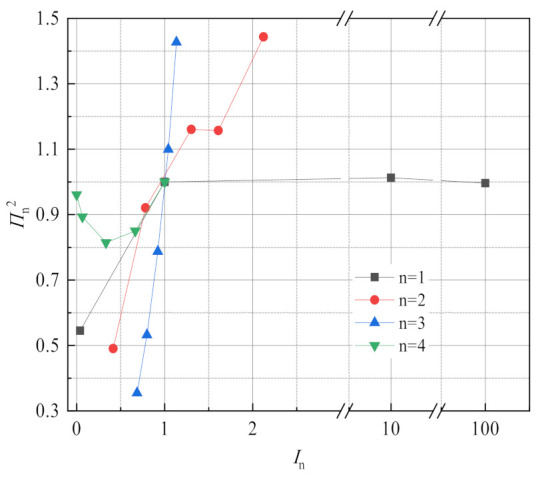
Sensitivity analysis of flue gas waste heat recovery.

This experiment focuses on the effects of flue gas temperature, flue gas flow, negative pressure airflow and membrane pore size on flue gas water and waste heat recovery characteristics, but flue gas water recovery characteristics and waste heat recovery characteristics have different sensitivity to fluctuations of different factors. Taking membrane pore size 10 nm, flue gas flow 10.33 L/min, flue gas inlet temperature 56.31 °C, negative pressure airflow 15 L/min as the reference operating conditions, Figure 20 shows the change of the dimensionless dependent variables of the flue gas water recovery characteristics with the dimensionless independent variables of different factors. In addition to the 0.4 nm membrane pore size, it can be see that the sensitivity of flue gas water recovery characteristics to different factors in descending order is flue gas inlet temperature, negative airflow, flue gas flow, and membrane pore size. In addition, taking a membrane pore size of 10 nm, flue gas flow 10.33 L/min, flue gas inlet temperature 56.31 °C, negative pressure airflow 15 L/min as reference conditions, Figure 21 shows the change of the dimensionless dependent variables of the flue gas waste heat recovery characteristics with the dimensionless independent variables of different factors, which is different from the water recovery characteristics of flue gas. In addition to the 0.4 nm membrane pore size, it can be seen that the sensitivity of flue gas waste heat recovery to different factors in descending order is flue gas inlet temperature, flue gas flow, negative pressure airflow, and membrane pore size.

### 3.4. Latent/Sensible Heat Recovery Characteristics

**Figure 22:**
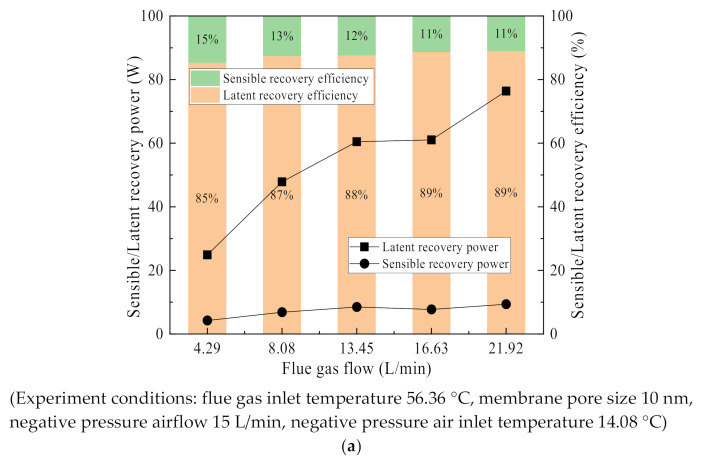

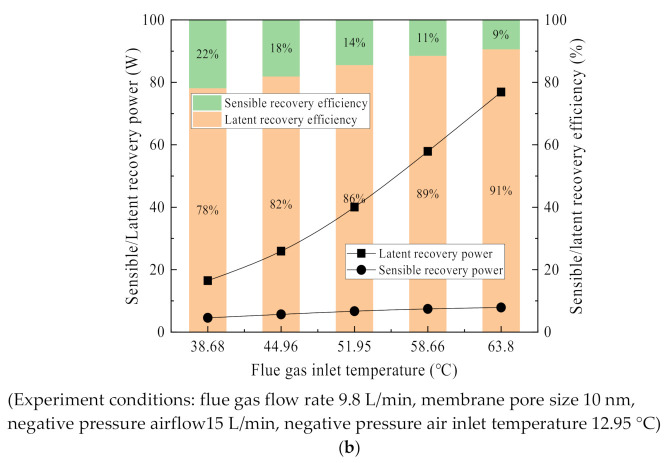
Sensible heat/latent heat recovery characteristics from flue gas. (**a**) Latent/sensible heat recovery power/proportion vs. Flue gas flow. (**b**) Latent/sensible heat recovery power/proportion vs. Flue gas inlet temperature.

There is a large amount of water in the form of water vapor in the low temperature wet saturated flue gas, so the flue gas waste heat recovery can be divided into sensible heat recovery and latent heat recovery. As shown in Figure 22, the flue gas latent heat recovery power increases significantly with flue gas temperature and flue gas flow, while the flue gas sensible heat recovery power increases slowly. At the same time, latent heat recovery is the main heat recovery power, accounting for up to 90%. The sensible heat recovery is relatively small, accounting for only about 10%. It was also confirmed that waste heat recovery is dominated by latent heat recovery in literature [37,39], but the latent heat proportion is higher in this paper. The higher the flue gas temperature, the larger the latent heat proportion of water vapor. In this paper, negative pressure air is used as the cooling medium, and the temperature difference is small between the flue gas inlet and outlet in the membrane module, resulting in a larger latent heat recovery proportion. As the flue gas flow rate or flue gas temperature increases, both the sensible heat and latent heat recovery increase, but the proportion of sensible heat decreases. Because of the higher flue gas temperature, the moisture content in the flue gas is higher, and the latent heat per unit temperature difference accounts for a relatively large amount. As shown in Figure 22b, with the flue gas temperature rises, compared with the sensible heat recovery power, the latent heat recovery power has increased significantly, and the proportion of sensible heat recovery power has been reduced from 22% to 9%. In this paper, the variation of latent heat recovery with flue gas inlet temperature or flue gas flow is exactly the opposite of the findings in the literature [37] because the flue gas in this paper is wet saturated, while the relative humidity of the flue gas is only 70% in the literature [37], which results in a lower dew point temperature.

### 3.5. Negative Pressure Air Characteristics

**Figure 23:**
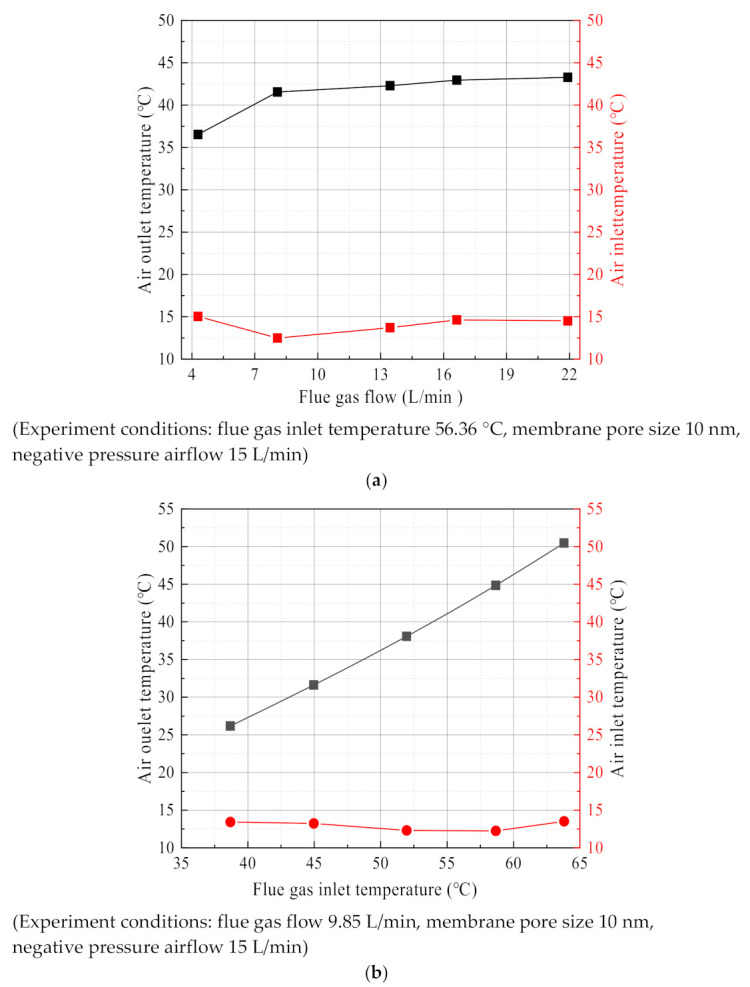
The inlet/outlet temperature of negative pressure air. (**a**) The temperature of negative pressure air vs. Flue gas flow. (**b**) The temperature of negative pressure air vs. flue gas inlet temperature.

The negative pressure air in the ceramic membrane module tube side is used as the cooling medium, and the temperature is significantly increased after absorbing the waste heat from the flue gas. As shown in Figure 23, the negative pressure air outlet temperature is significantly higher than the negative pressure air the inlet temperature. At this time, the negative pressure air in the ceramic membrane absorbs a large amount of flue gas waste heat. As shown in Figure 23a, the negative pressure air outlet temperature increased slightly with the increase of flue gas flow, from 36.5 °C to 43.2 °C. While in Figure 23b, the negative pressure air outlet temperature continued to increase rapidly with the increase of flue gas temperature, from 26.2 °C up to 50.5 °C. Although the increase in flue gas flow increases the input of flue gas waste heat in the ceramic membrane module, the flue gas temperature is relatively stable. The heat exchange temperature difference in the heat transfer process is basically unchanged, resulting in a slight increase in the outlet temperature of negative pressure air. However, increasing the flue gas temperature, on the one hand, increases the input of the flue gas waste heat in the ceramic membrane module. On the other hand, there is a significant increase in the heat exchange temperature difference of the ceramic membrane module, so the negative pressure air outlet temperature changes more with the flue gas temperature.

**Figure 24:**
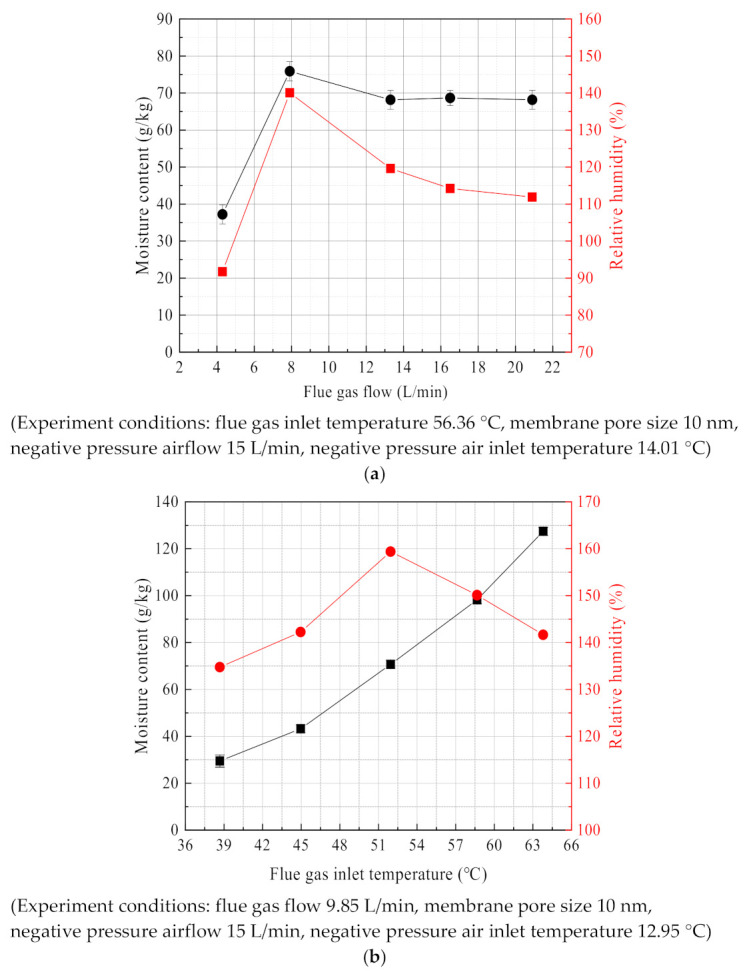
Humidity characteristics of the negative pressure air outlet. (**a**) Moisture content/relative humidity vs. Flue gas flow. (**b**) Moisture content/relative humidity vs. flue gas inlet temperature.

As shown in Figure 24a, with the increase of the flue gas flow, the negative pressure air moisture content at the outlet of the membrane module tube side increases first and then tends to be flat, which is consistent with the change trend of the water recovery rate. On the one hand, the flue gas flow increasing helps to increase the water recovery rate from flue gas. On the other hand, it will also cause the moisture in the flue gas to stay in the membrane for a short time and cannot be transmitted to the negative pressure air. While the relative humidity first increases and then decreases, because the relative humidity is mainly affected by the negative pressure air outlet temperature and moisture content. As the flue gas flow increases, the negative pressure air outlet temperature continues to increase, and the negative pressure air outlet moisture content eventually stabilizes, so the relative humidity will appear to change—a different trend from the moisture content.

Figure 24b shows that the influence characteristics of flue gas temperature on the moisture content. As the flue gas temperature increases, the moisture content of the negative pressure air outlet continuously increases because the same flue gas residence time and greater partial pressure of water vapor in the flue gas, can increase the driving force for mass transfer. As the flue gas temperature rises, the relative humidity of the negative pressure air outlet increases first and then decreasing. Although the moisture content of the negative pressure air continues to increase, the negative pressure air outlet temperature increases faster. At this time, the temperature has a more impact on the relative humidity.

The relative humidity of negative pressure air is determined by the moisture content in the saturated state and the actual moisture content together. The saturation moisture content is determined by the negative pressure air outlet temperature, while the actual moisture content is determined by the water recovery rate. Figure 23 shows the relative humidity above 100%, because the actual moisture content is higher than the saturation moisture content. By this time, the negative pressure air reaches saturation. The condensate cannot continue to evaporate into water vapor but be carried out from the membrane module as liquid droplets by the negative pressure air. Thus, the relative humidity will be higher than 100%.

## 4. Conclusions

This paper experimentally studies the water and waste heat recovery from flue gas by the ceramic membrane condenser with the help of negative pressure air, and considers the reuse of flue gas waste heat. Through detailed analysis of the experimental results, the key factors that affect the characteristics of flue gas water and waste heat recovery are clarified, the composition of flue gas waste heat recovery is analyzed, and the negative pressure air humidity parameters at the outlet of the ceramic membrane module tube side are discussed.

(1) The flue gas temperature is the most sensitive factor related to the water and waste heat recovery characteristics from the flue gas. Increasing the flue gas temperature helps to increase the water recovery rate and heat recovery power from flue gas, but the water recovery efficiency and heat recovery efficiency will increase first and then decrease. Increasing the flue gas flow can increase the heat recovery power, but it will not continue to increase the water recovery rate. At the same time, it will reduce the water recovery efficiency and heat recovery efficiency. When the negative gas airflow is maximum or zero, the water and heat recovery performance are better, but the high vacuum degree has the risk of infiltration of non-condensable gas. Except for the 0.4 nm ceramic membrane, the membranes with different pore sizes have little effect on the water and waste heat recovery characteristics from flue gas.
(2) The waste heat recovery of flue gas is dominated by latent heat recovery of water vapor, accounting for 80% and above. The negative pressure air is heated and humidified by the ceramic membrane condenser, and the negative pressure air outlet temperature has a significant promote. Compared with the high vacuum, increasing the negative pressure airflow has more practical application possibilities. Thus, a pore size of 0.4 nm in ceramic membranes is too small, and is not suitable for water and waste heat recovery from flue gas.

## Figures and Tables

**Table 1:** Structural parameters of the ceramic membrane module.

Project	Unit	Ceramic Membrane				Shell
		1	2	3	4	
Average pore size	nm	0.4	10	100	1000	/
Structure	/	Asymmetric			Symmetry	
Coating	/	Outer coating			/	/
Material	/	Alumina				AISI 316L
Length	mm	790	790	790	790	800
Outer diameter	mm	12				22
Inter diameter	mm	8				20
Porosity	%	31.56%	33.62%	33.86%	34.12%	/
Outer surface area	cm^2^	297.67				/
Inner surface area	cm^2^	198.45				/
Flow area	cm^2^	0.50				2.01

Note: AISI 316L-stainless material. “/” means no content.

**Table 2:** Selection of experimental parameters.

Parameter	Unit	Value	Parameter	Unit	Value
Flue gas temperature	°C	38–64	Negative pressure air temperature	°C	12–14
Flue gas flow	L/min	4–22	Negative pressure airflow	L/h	0–15

**Table 3:** Instrument parameters.

Instrument	Model	Range	Uncertainty
Metal rotor flowmeter	CGYL-LZ-25	0–30 L/min	1.0%
Glass rotor flowmeter	LWGY-4-C-10	0–20 L/min	1.0%
Temperature transmitter	SWB-B	0–100 °C	0.25%
Pressure transmitter	CGYL-202	−50–50 kPa	0.25%

## Data Availability

Not applicable.
